# CsCIPK11-Regulated Metalloprotease CsFtsH5 Mediates the Cold Response of Tea Plants

**DOI:** 10.3390/ijms24076288

**Published:** 2023-03-27

**Authors:** Taimei Di, Yedie Wu, Jing Peng, Jie Wang, Haoqian Wang, Mingming He, Nana Li, Xinyuan Hao, Yajun Yang, Dejiang Ni, Lu Wang, Xinchao Wang

**Affiliations:** 1 Key Laboratory of Biology, Genetics and Breeding of Special Economic Animals and Plants, Ministry of Agriculture and Rural Affairs, National Center for Tea Plant Improvement, Tea Research Institute, Chinese Academy of Agricultural Sciences, 9th South of Meiling Road, Hangzhou 310008, China; 2 College of Horticulture & Forestry Sciences, Huazhong Agricultural University, Wuhan 430070, China; 3 College of Horticulture, Fujian Agriculture and Forestry University, Fuzhou 350002, China

**Keywords:** cold, CsCIPK11, CsFtsH5, photosynthetic activity, tea plant

## Abstract

Photosystem II repair in chloroplasts is a critical process involved in maintaining a plant’s photosynthetic activity under cold stress. FtsH (filamentation temperature-sensitive H) is an essential metalloprotease that is required for chloroplast photosystem II repair. However, the role of FtsH in tea plants and its regulatory mechanism under cold stress remains elusive. In this study, we cloned a *FtsH* homolog gene in tea plants, named *CsFtsH5*, and found that CsFtsH5 was located in the chloroplast and cytomembrane. RT-qPCR showed that the expression of *CsFtsH5* was increased with leaf maturity and was significantly induced by light and cold stress. Transient knockdown *CsFtsH5* expression in tea leaves using antisense oligonucleotides resulted in hypersensitivity to cold stress, along with higher relative electrolyte leakage and lower *Fv/Fm* values. To investigate the molecular mechanism underlying *CsFtsH5* involvement in the cold stress, we focused on the calcineurin B-like-interacting protein kinase 11 (*CsCIPK11*), which had a tissue expression pattern similar to that of *CsFtsH5* and was also upregulated by light and cold stress. Yeast two-hybrid and dual luciferase (Luc) complementation assays revealed that CsFtsH5 interacted with CsCIPK11. Furthermore, the Dual-Luc assay showed that CsCIPK11-CsFtsH5 interaction might enhance CsFtsH5 stability. Altogether, our study demonstrates that *CsFtsH5* is associated with *CsCIPK11* and plays a positive role in maintaining the photosynthetic activity of tea plants in response to low temperatures.

## 1. Introduction

Light and temperature are two critical environmental factors regulating physiological and developmental processes in plants. Exposure to adverse environmental conditions, including extreme light and temperature, can lead to oxidative stress in plants. The organelle that is most vulnerable to oxidative damage is the chloroplast, the proper function of which is a prerequisite for the normal growth and development of plants. Chloroplasts are semi-autonomous organelles assembled with functional multi-subunit photosynthetic complexes, including photosystem II (PSII) and photosystem I (PSI) [1,2]. Photodamage to the PSII complex is an unavoidable process under stress conditions. Thus, quality control of the PSII complex is critical for maintaining photosynthetic activity in the presence of excessive reactive oxygen species (ROS) [3,4]. Photodamage primarily affects the PSII reaction center protein D1. The rapid and selective removal of photodamaged D1 is a critical step in the reactivation and reassembly of PSII, thus ensuring the maintenance of photosynthetic activity. In fact, plants have developed effective mechanisms for degrading damaged D1 [5,6]. Research indicates that the thylakoid membrane-bound FtsH metalloprotease plays a central role in the degradation of the damaged D1 protein [7,8].

FtsH is a conserved membrane-anchored metalloprotease that contains an ATPase domain and a catalytic zinc-binding site [9]. FtsH was first identified in prokaryotes [10] and then demonstrated in the chloroplasts and mitochondria of eukaryotic cells [11,12]. To date, twelve *FtsH* genes have been identified in arabidopsis (*Arabidopsis thaliana* (L.) Heyn.) [13]. Functional redundancy and sequence homology analyses show that FtsH in thylakoid membranes are present in hexameric heterocomplexes, consisting of four major isomers divided into two major isoforms: Type A (FtsH2 and FtsH8) and Type B (FtsH1 and FtsH5) [13,14]. FtsH2 and FtsH5 are the dominant subunits, and mutations in either cause impaired chloroplast development and maintenance, resulting in leaf variegation phenotypes [13]. Additionally, arabidopsis *ftsh5* and *ftsh2* mutants exhibited susceptibility to photooxidative stress with concomitant excessive ROS accumulation [13,15], and *ftsh2* mutants exhibited susceptibility to 10 °C low temperature and failure to control PSII quality [16]. 

Although chloroplast FtsH5 and FtsH2 are predominantly responsible for PSII repair and photoprotection, it remains unclear how their functions are modulated. Several reports have suggested that proteins associated with the thylakoid membranes are involved in modulating FtsH proteases. In arabidopsis, a null mutation in THYLAKOID FORMATION (*thf1*) exhibited a leaf variegation phenotype, and the *thf1* mutant had reduced FtsH proteases [17,18,19]. GTPase AtEngA interacts with FtsH complexes and represses FtsH activities [20]. AtFIP, a small thylakoid-anchored protein, interacts with type A FtsH subunits and may negatively modulate FtsH activity [21]. In addition, studies have suggested that FtsH, especially FtsH5, can be phosphorylated in a calcium-dependent manner in the thylakoid, which may partially influence the stability, proteolytic activity, and development of the FtsH complex [22]. AtCIPK13, a calcineurin B-like (CBL)-interacting protein kinase located in the chloroplast, has been speculated to be a candidate kinase involved in calcium-dependent signal transduction in chloroplasts [23,24]. However, the specific kinase responsible for the calcium-dependent protein phosphorylation of FtsH has not been characterized in detail. 

FtsH proteases have been widely studied in multiple plant species; however, they are not well studied in tea plants (*Camellia sinensis* (L.) *O. Kuntze*). Being a low temperature-sensitive economic crop, a tea plant’s ability to tolerate cold stress has become a vital research topic in the context of climate change. We have previously reported that the calcium signaling pathway and the family of CIPK kinases play a vital role in the cold response of tea plants [25,26]. In the current study, we aimed to determine if *CsFtsH5*, the ortholog of *AtFtsH5*, was upregulated by light and low temperature. Here, we report that CsFtsH5 was involved in the cold tolerance of tea plants and interacted with CsCIPK11, which enhanced the accumulation of CsFtsH5 protein. We surmise that the CsFtsH5 regulation by CsCIPK11 is a key process for adjusting chloroplast functions to adapt to environmental needs. These findings will serve as a foundation for further studies on calcium-dependent phosphorylation of thylakoid proteins. 

## 2. Results

### 2.1. Sequence and Phylogenetic Analyses of CsFtsH5 in Tea Plants

**Figure 1:**
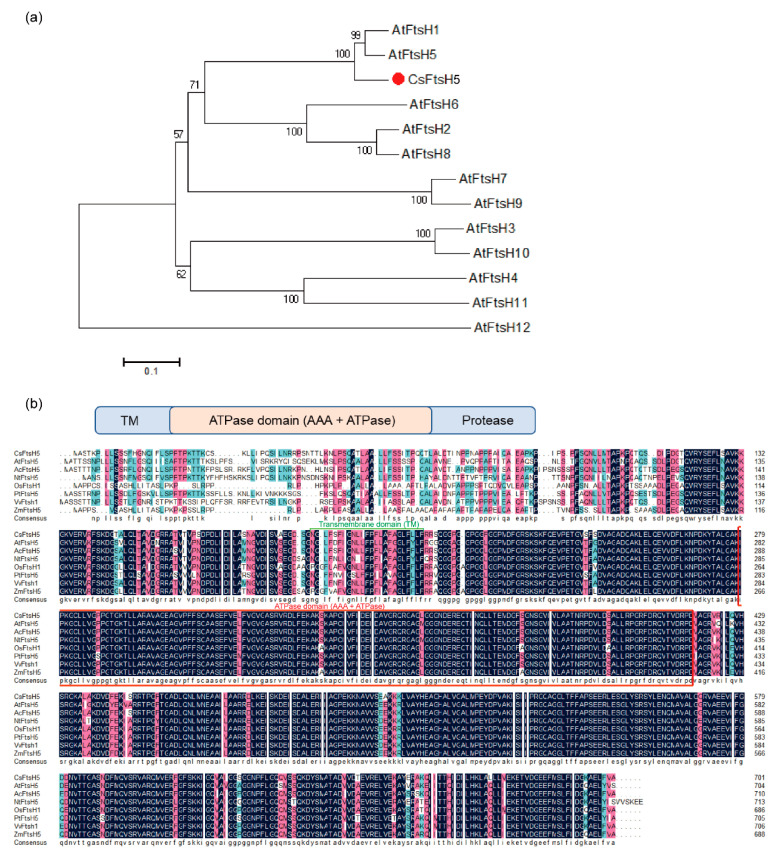
Phylogenetic analyses and protein sequence alignment of CsFtsH5. (**a**) Neighbor-joining phylogenetic tree of CsFtsH5 and AtFtsHs. The red dot indicates CsFtsH5. (**b**) Bioinformatic features of CsFtsH5 domains and multiple sequences alignment of CsFtsH5. At: *Arabidopsis thaliana*; Pt: *Populus trichocarpa*; Nt: *Nicotiana tabacum*; Zm: *Zea mays L.*; Vv: *Vitis vinifera*; Os: *Oryza sativa*; Ac: *Actinidia Chinensis*.

The gene *CsFtsH5* was named according to its arabidopsis homolog and was cloned from the leaf cDNA of the ‘Longjing 43’ tea plant. *CsFtsH5* encodes a protein of 701 amino acid residues and has a deduced molecular mass of 75.13 kDa. To investigate the evolutionary relationship of CsFtsH5, a phylogenetic tree was constructed using the amino acid sequences of arabidopsis FtsHs. As expected, AtFtsH5 and AtFtsH1 were classified into the same group, and CsFtsH5 shared the closest relationship with AtFtsH5 (85.2% homology) rather than AtFtsH1 (83.9% homology) (Figure 1a). Similar to the typical bioinformatic characteristics of FtsH [9,27], FtsH5 consists of a transmembrane domain at the N-terminus, followed by a highly conserved AAA^+^ ATPase domain, which is responsible for the binding and hydrolysis of ATP, and a protease domain (Figure 1b). According to the result of multiple sequence alignment, CsFtsH5 shared a high similarity in amino acid sequence with formerly reported FtsH5s in other species in the AAA^+^ ATPase and protease domains, yet they displayed variation at the N-terminal domain (Figure 1b), indicating that CsFtsH5 may undertake a similar role to other homologous proteins in mediating protein degradation.

### 2.2. Expression Pattern and Sub-Cellular Localization of CsFtsH5

**Figure 2:**
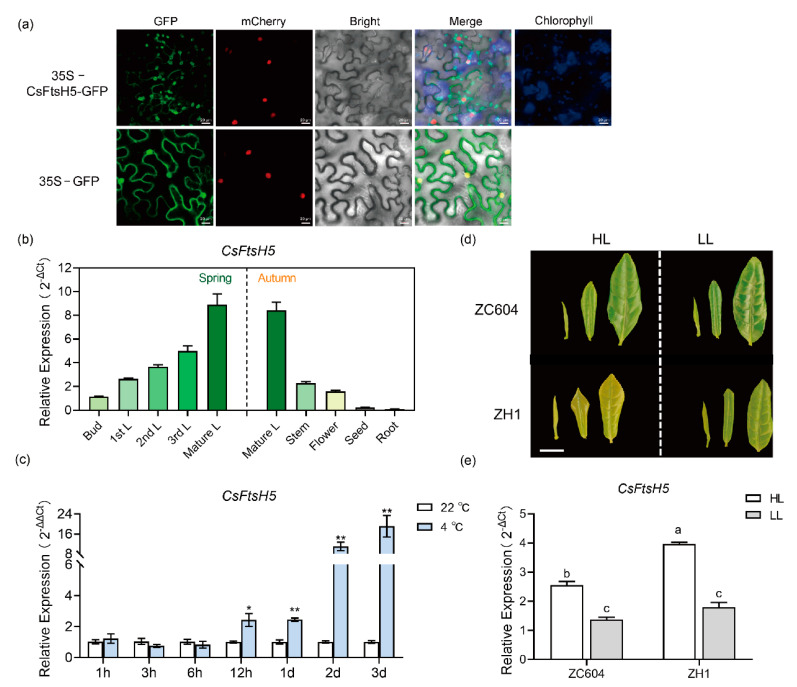
Expression profiles and sub-cellular localization of *CsFtsH5*. (**a**) Sub-cellular localization of CsFtsH5 protein in H2B-RFP transgenic *Nicotiana benthamiana* leaves. The empty 35S-GFP was a positive control. Nuclei are indicated by the H2B-RFP (red), and the chloroplasts are shown in blue. Scale bar = 20 µm. (**b**)The expression patterns of *CsFtsH5* in tea plant tissues, including bud, 1st L, 2nd L, 3rd L, mature L, stem, flower, seed, and root. Samples were collected on April, 2020, and October, 2020. (**c**) The *CsFtsH5* expression in tea plants under 22 °C and 4 °C treatments for 1, 3, 6, and 12 h, as well as 1, 2, and 3 d. Data are shown as the mean ± SEM (*n* = 3). Asterisks indicate significant differences according to *t*-tests (* *p* < 0.05, ** *p* < 0.01). (**d**) The phenotype of 2 leaves and 1 bud of ZC604 and ZH1 under 100% and 10% sunlight conditions. Scale bar = 1 cm. (**e**) The expression patterns of *CsFtsH5* in leaves of the green-leaf cultivar of ZC604 cultivar and the yellow-leaf cultivar of ZH1 under 100% and 10% sunlight conditions. Means of three replicates and standard errors are presented; different letters above the column indicate a significant difference at *p* < 0.05 using LSD’s test. Values in (**b**,**e**) are expressed relative to the expression levels of the reference gene using the formula 2^−ΔCt^. Values in (**c**) are expressed relative to the expression levels of the 22 °C group using the formula 2^−ΔΔCt^. *CsPTB* was used as a reference gene in expression analysis.

To explore the subcellular localization and potential function of the CsFtsH5 protein, the 35S-GFP (green fluorescent protein) construct fused with CsFtsH5 was generated and transiently expressed in H2B-RFP transgenic *N. benthamiana* leaves [28]. The vector control, which contained GFP alone, was found throughout the cell, whereas the CsFtsH5-GFP protein was visible in the chloroplast and membrane, which implied the potential role of CsFtsH5 in leaf photosynthesis (Figure 2a). The expression pattern of *CsFtsH5* was analyzed in different organs of tea plants. RT-qPCR results showed that *CsFtsH5* was detected in all selected tissues, including the first leaf (1st L), second leaf (2nd L), third leaf (3rd L), and mature leaf (mature L) sampled in the spring, and the mature L, stem, flower, seed, and root sampled in the autumn. The levels of *CsFtsH5* expression differed among the tissues. *CsFtsH5* was highly expressed in leaves, whereas lower expression was detected in roots, flowers, and seeds. Interestingly, the increased expression of *CsFtsH5* occurred with leaf maturity (Figure 2b). These results suggest that *CsFtsH5* is involved in tea plant growth and development, particularly important for the development of leaves in tea plants.

Given that FtsH is required for chloroplast photosystem II repair, which is a critical process in maintaining a plant’s photosynthetic activity under cold and light stress, we analyzed the response pattern of *CsFtsH5* in tea plants to cold stress. RT-qPCR results showed that *CsFtsH5* expression was gradually elevated after 12 h under the 4 °C treatment and peaked at the 3 d time point (18.8-fold; Figure 2c). Further, we analyzed the expression pattern of *CsFtsH5* in tea leaves under different light intensity treatments (100% sunlight, HL (high light); 10% sunlight, LL (low light)). RT-qPCR results showed that *CsFtsH5* was significantly induced by light in both tea cultivars, i.e., the green-leaf cultivar ‘ZC604’ (‘Zhongcha 604’) and the chlorophyll-deficient cultivar ‘ZH1’ (‘Zhonghuang 1’) [29], whose leaves exhibited yellow color under high-light conditions and green under low-light conditions (Figure 2d). Interestingly, the expression level of *CsFtsH5* in ‘ZH1’ was higher than that in ‘ZC604’ under HL conditions (Figure 2e), indicating that *CsFtsH5* expression is likely related to chloroplast function. These results suggest that *CsFtsH5* is involved in light and low-temperature responses in tea plants. 

### 2.3. CsFtsH5 Down-Regulation Causes Tea Leaf Hypersensitivity to Cold Stress

**Figure 3:**
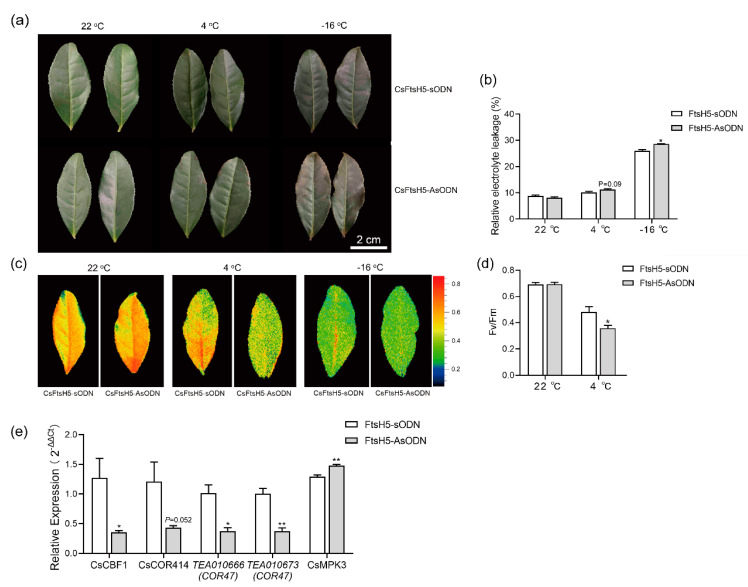
*CsFtsH5*-AsODN tea plants showed hypersensitivity to cold stress. (**a**) Phenotypes of *CsFtsH5*-AsODN and *CsFtsH5*-sODN plants treated with cold stress (4 °C for 5 d followed by 22 °C recovery for 2 d) and freezing stress (22 °C for 4.5 d followed by −16 °C for 12 h, then 22 °C recovery for 2 d). Control group was grown at 22 °C for 7 d. Scale bar = 2 cm. (**b**–**d**). Relative electrolyte leakage, chlorophyll fluorescence images, and *Fv/Fm* value of *CsFtsH5*-AsODN and *CsFtsH5*-sODN plants treated with low temperature. (**e**) The relative expression of *CsCBF* and *CsCOR* in *CsFtsH5*-AsODN and *CsFtsH5*-sODN plants under 4 °C low temperature. Mean and standard deviation values were obtained from at least three independent experiments. The asterisks indicated significantly different values between *CsFtsH5*-AsODN and *CsFtsH5*-sODN plants according to *t*-tests (* *p* < 0.05, ** *p* < 0.01).

To examine the role of *CsFtsH5* in the cold stress responses of the tea plant, we next effectively and transiently suppressed *CsFtsH5* expression (Figure S1) by using the gene-specific antisense oligonucleotides (AsODN) method, as previously described by Zhao et al. [30]. There were no obvious differences in plant phenotypes between *CsFtsH5*-AsODN and *CsFtsH5*-sODN plants under normal conditions (Figure 3a). However, under cold and freezing stress, the blade edge of *CsFtsH5*-AsODN leaves was more damaged than that of *CsFtsH5*-sODN leaves (Figure 3a). We then measured the *Fv/Fm* values and the relative electrolyte leakage (REL) of the tea leaves before and after low-temperature stress. The *CsFtsH5*-AsODN plants exhibited significantly lower *Fv/Fm* values and relatively higher REL values (*p* = 0.09) than the *CsFtsH5*-sODN plants under cold stress (Figure 3b–d). Further, the REL value of *CsFtsH5*-AsODN plants was significantly higher than that of *CsFtsH5*-sODN plants under freezing stress (Figure 3b). These results suggest that down-regulation of *CsFtsH5* leads to enhanced cold sensitivity in tea plants.

The CBF pathway plays a critical role in plant cold response. Thus, we next examined the expression pattern of the genes involved in the CBF pathway, including the positive regulators of *CsCBF1*, *CsCOR414*, and *CsCOR47*, and the negative regulator of *CsMPK3* in *CsFtsH5*-AsODN and *CsFtsH5*-sODN plants under cold stress [31]. The results showed that compared with *CsFtsH5*-sODN leaves, the transcript abundance of *CsCBF1*, *CsCOR414*, and *CsCOR47* was significantly repressed in *CsFtsH5*-AsODN tea leaves under cold stress, whereas *CsMPK3* was highly expressed in *CsFtsH5*-AsODN (Figure 3e). Taken together, these data indicate that *CsFtsH5* plays a positive role in regulating cold-regulated gene expression in tea plants.

### 2.4. Interaction between CsFtsH5 and CsCIPK11

**Figure 4:**
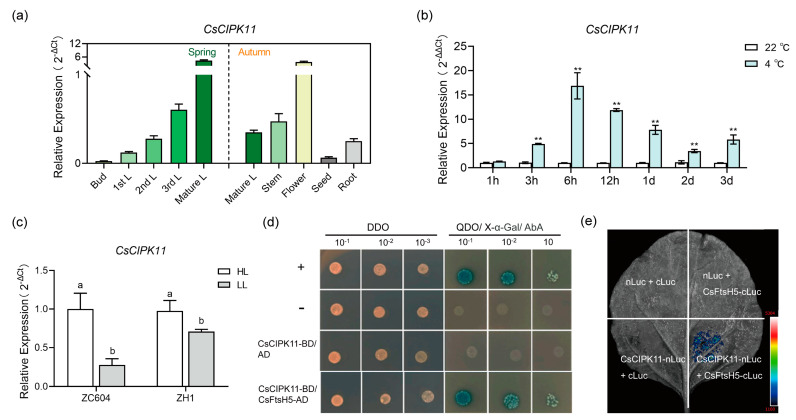
CsFtsH5’s interaction with CsCIPK11. (**a**) The expression patterns of *CsCIPK11* in tea plant tissues, including bud, 1st L, 2nd L, 3rd L, mature L, stem, flower, seed, and root. Samples were collected on April 2020 and October 2020. (**b**) The *CsCIPK11* expression in tea plants under 22 °C and 4 °C treatments for 1, 3, 6, and 12 h, as well as 1, 2, and 3 d. Data are shown as the mean ± SEM (*n* = 3). Asterisks indicate significant differences according to *t*-tests (** *p* < 0.01). (**c**) The expression patterns of *CsCIPK11* in leaves of ZC604 cultivar and ZH1 cultivar under 100% and 10% sunlight conditions. Means of three replicates and standard errors are presented; different letters above the column indicate significant difference at *p* < 0.05 using LSD’s test. Values in (**a**,**c**) are expressed relative to the expression levels of reference gene using formula 2^−ΔCt^. Values in (**b**) are expressed relative to the expression levels of 22 °C group using formula 2^−ΔΔCt^. *CsPTB* was used as a reference gene in expression analysis. (**d**) Y2H assay of pGBKT7-CsCIPK11 and pGADT7-CsFtsH5 in AH109 strain. DDO, SD/−Leu/−Trp; QDO, SD/−Leu/−Trp/−His/−Ade; X, X-α-gal. pGBKT7-53 and pGADT7-T acted as the positive control, while pGBKT7-lam and pGADT7-T served as the negative control. (**e**) Luciferase complementation imaging analysis of CsCIPK11-nLuc and CsFtsH5-cLuc in *Nicotiana benthamiana* leaves. CsCIPK11-nLuc with cLuc, nLuc with CsFtsH5-cLuc, and nLuc with cLuc served as controls.

Next, we investigated the molecular mechanism underlying *CsFtsH5* involvement in the cold stress response. Considering our previous research involving the CsCIPK family, we focused on *CsCIPK11*, which showed a tissue expression pattern similar to that of *CsFtsH5* (Figure 4a). Expression analysis showed that *CsCIPK11* was highly expressed in mature L and flower and exhibited lower expression in bud and seed. Meanwhile, consistent with *CsFtsH5*, the expression level of *CsCIPK11* was positively related to the maturity of the tea leaf (Figure 4a). Further, the expression of *CsCIPK11* was also induced by cold stress and light signals (Figure 4b,c). Exposed to 4 °C low temperature, *CsCIPK11* in young leaf was rapidly and significantly induced at the 3 h point, and its highest expression appeared at 6 h (Figure 4b). In addition, *CsCIPK11* was significantly induced by light in ‘ZC604’ and ‘ZH1’ (Figure 4c).

Furthermore, the yeast two-hybrid (Y2H) assay showed that cells co-transformed with CsFtsH5 and CsCIPK11 grew and turned blue on QDO (SD/−Leu/−Trp/−His/−Ade; X-α-gal) medium (Figure 4d), indicating that the interaction between CsCIPK11 and CsFtsH5 occurred in yeast. Subsequently, the luciferase complementation imaging (LCI) assay showed that an obvious luminescence signal appeared only in the regions of leaves co-expressing CsFtsH5-cLuc and CsCIPK11-nLuc, whereas the Luc signal was not detected when the empty vector was expressed in the same proportions (Figure 4e). These data are strongly suggestive of an interaction between CsFtsH5 and CsCIPK11.

### 2.5. CsCIPK11 May Contribute to CsFtsH5 Protein Stability

**Figure 5:**
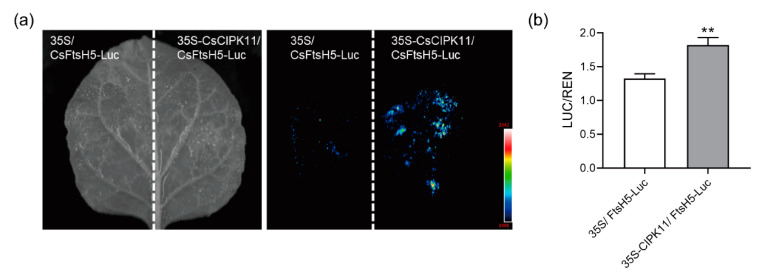
CsCIPK11 may stabilize the CsFtsH5 protein. (**a**) Fluorescence observations in *Nicotiana benthamiana* leaves that expressed 35S-CsCIPK11/35S-CsFtsH5-Luc and 35S-empty vector/35S-CsFtsH5-Luc. (**b**) Relative LUC/REN activity measurements in dual-Luciferase assays. The relative LUC/REN value is the average of six biological replicates. Error bars indicate the SEM of six biological replicates. Asterisks indicate significant differences according to *t*-tests (** *p* < 0.01).

In order to explore the molecular mechanism of the interaction, we further investigated whether the interaction between CsCIPK11 and CsFtsH5 affected the stability of the latter. We co-expressed 35S-CsCIPK11 and an empty vector with 35S-CsFtsH5-Luc in *N. benthamiana* leaves. The fluorescence signal was stronger in leaves co-expressing 35S-CsCIPK11 and 35S-CsFtsH5-Luc than in leaves co-expressing the 35S-empty vector and 35S-CsFtsH5-Luc (Figure 5a). Further, the relative LUC/REN activity measurements also support the fluorescence signal results (Figure 5b). Thus, our data indicate that CsCIPK11 interacted with CsFtsH5 and increased its accumulation in vivo. CsCIPK11-CsFtsH5 interaction may enhance the stability of CsFtsH5.

## 3. Discussion

The chloroplast is the most susceptible organelle to low-temperature-induced oxidative damage. FtsH metalloproteases are identified as the master regulators in maintaining the photosynthetic activity of chloroplast [8,32]. Considering the growth and development of tea plants are limited by low temperatures, a better understanding of the role of *CsFtsH* in cold stress responses can provide meaningful information for adversity-resistance breeding in tea plants.

### 3.1. Metalloprotease CsFtsH5 of Tea Plant Is Homologous to FtsH5

FtsH proteases are membrane-bound metalloproteases that are present in eubacteria, animals, and plants. Here, we isolated and identified *CsFtsH5* from tea plants. Sequence and phylogenetic analyses showed that CsFtsH5 is a protein homologous to FtsH5 and contains highly conserved ATPase and protease domains (Figure 1a,b), indicating that FtsH is an evolutionarily conserved protein across various species. The ATPase domain is involved in protein degradation, and its central pore allows substrate proteins to unfurl and enter the proteolytic chamber, where polypeptide chains are gradually broken down into short peptides [33]. Thus, we speculate that CsFtsH5 may be involved in protein degradation, especially D1 degradation, similar to AtFtsH2 in arabidopsis and PvFtsH2 in common beans [7,16,34,35]. The role of CsFtsH5 in D1 degradation requires further investigation. Interestingly, while AtFtsH5 is located only in the chloroplast [15], we found that CsFtsH5 was located both in the chloroplast and membrane (Figure 2a). This observation raises the possibility of different roles played by FtsH5 between arabidopsis and tea plants. Loss of *AtFtsH5* caused leaf variegation in arabidopsis [15]. *CsFtsH5* was primarily expressed in leaves, and the expression level was positively correlated with leaf maturity (Figure 2b), suggesting that *CsFtsH5* is potentially involved in leaf development in tea plants. Further, we found that similar to *AtFtsH5* [15], *CsFtsH5* is also a light-responsive gene (Figure 2d,e). Noticeably, in response to light exposure, yellow leaves displayed greater *CsFtsH5* expression compared to green leaves (Figure 2e). These results are consistent with those obtained by a previous study on chrysanthemums, which reported that *CmFtsH* expression is regulated by light and is higher expressed in the yellow leaf tissue [36]. Thus, such responses indicate a feedback regulation in which chrysanthemun and tea plants enter an altered physiological status followed by activation of *CmFtsH* and *CsFtsH5*, respectively.

### 3.2. Repressing CsFtsH5 Facilitated the Cold Sensitivity of Tea Plants

FtsH proteases are necessary for maintaining the quality of membrane protein and preventing chloroplast damage from cold and heat [16,37], which coincided with the location of CsFtsH5 in the chloroplast and membrane (Figure 2a). We noticed a significant induction of *CsFtsH5* expression by low-temperature treatment (Figure 2c), indicating that *CsFtsH5* is responsive to low temperatures in tea plants. 

When arabidopsis is exposed to high temperatures, the photosynthetic capability of the *ftsh11* mutant is greatly reduced. Further, the AtFtsH11 protease alleviates the thermotolerance damage of the photosynthesis apparatus [38]. Compared with the wild type, *ftsh2* mutants exhibit a drastic decline in PSII activity and a significant cell death response under low-temperature and high-light-intensity conditions [16]. Those results showed that *FtsH* participated in temperature stress by regulating chloroplast activity. Our study also characterized the role of *CsFtsH5* in response to low temperatures. Downregulation of *CsFtsH5* enhanced cold sensitivity (Figure 3), suggesting that *CsFtsH5* protected the tea plant from low-temperature stress, likely by maintaining the PSII activity, as indicated by the *Fv/Fm* results. In addition, the downregulating of *CsFtsH5* in tea leaves suppressed the CBF-mediated cold response pathway (Figure 3e). We speculate that suppressing *CsFtsH5* in tea leaves induced PSII damage and oxidative stress in the chloroplast, which may influence the CBF pathway and plant cold tolerance. However, the underlying mechanism needs to be further explored in future studies.

### 3.3. Regulation of CsFtsH5 by CsCIPK11 Likely Mediates the Cold Response of Tea Plants

Several studies have reported the molecular mechanisms underlying FtsH regulation in plants. For example, a soybean study reported that GmFtsH25 interacts with photosystem I light-harvesting complex 2 (GmLHCa2), which contributes to enhanced photosynthesis [39]. An arabidopsis study found that AtFtsH11 is a critical regulator of photosynthetic thermotolerance. AtFtsH11 interacted with the ATP synthase assembly factor BFA3, which degraded under high temperatures, leading to the adjustment of ATP synthase assembly in the chloroplast in response to heat stress [40]. Furthermore, AtTHF1 is necessary for AtFtsH to accumulate normally in the thylakoid membrane. AtFtsH proteases were reduced in the *thf1* mutant, leading to a leaf variegation phenotype [19]. AtEngA interacts with AtFtsH, and overexpression of *EngA* enhances FtsH stability, which engenders the dysfunction of FtsH protease activity in chloroplasts [20]. Therefore, proper turnover of FtsH proteases is crucial for their activity [20]. However, the detailed molecular mechanisms underlying FtsH5 regulation in plants remain unclear. 

Ca^2+^ is a vital secondary messenger in both plant development and early stress responses [41]. AtFtsH5 is phosphorylated in the thylakoid in a calcium-dependent manner, which possibly regulates FtsH stability in thylakoid membranes [22,24]. Extensive functional studies have demonstrated the broad involvement of CIPK family members in plant development and stress responses. For example, AtCIPK13 was found to be located in the chloroplast; therefore, AtCIPK13 was assumed to operate in chloroplasts and mediate calcium-dependent signal transduction pathways. However, detailed characterization has not yet been performed [22,23]. In agreement with this evidence, the current study found that the expression pattern of *CsCIPK11* was congruent with *CsFtsH5* in leaves with different maturities, as well as in response to cold and light (Figure 4a–c). Based on this result, we assessed the evidence for the interaction between CsFtsH5 and CsCIPK11 (Figure 4d,e) and found that CsCIPK11 may affect the stability of CsFtsH5 by interacting with it (Figure 5). Ca^2+^ is an important second messenger related to cold stimulus, and CIPK mediates the translation of Ca^2+^ information into downstream-specific responses. The response of *CsCIPK11* was quicker than that of *CsFtsH5* under cold stress (Figure 2c and Figure 4b). We speculated that CsCIPK11 might be the upstream regulator of CsFtsH5 under cold stress. Considering that the phosphorylation of thylakoid proteins is one of the most important regulatory mechanisms of photosynthesis, it is not yet clear if CsCIPK11 enhances the stability of CsFtsH5 protein through phosphorylation. Future studies should assess the role of CsCIPK11 and characterize whether phosphorylation of CsFtsH5 may be a means to alter its activity.

## 4. Materials and Methods

### 4.1. Plant Materials and Growth Conditions

Tea plant tissues, including bud, 1st L, 2nd L, 3rd L, mature L, stem, flower, seed, and root, were collected for RNA extraction from the 5-year-old ‘Longjing 43’ tea plant cultivar grown in the field at the Tea Research Institute of the Chinese Academy of Agricultural Sciences, Hangzhou, People’s Republic of China. Samples were collected in the spring (April 2020) and autumn (October 2020). To investigate *CsFtsH5* and *CsCIPK11* expression in response to cold stress, ‘Longjing 43’ tea plants were treated at 22 °C (control treatment) and 4 °C (experimental treatment) and maintained under the same photoperiod (12 h/12 h, light/dark). The first mature leaves were harvested for RNA extraction at 1, 3, 6, and 12 h, as well as 1, 2, and 3 d after treatment. To investigate *CsFtsH5* and *CsCIPK11* expression in response to light, 10-year-old tea plant cultivars of ‘ZC604’ and ‘ZH1’ with the same phenological phase were transferred to 100% and 10% sunlight conditions, and treatment was started from March 22 to April 8, 2019. Young shoots with two leaves and one bud were randomly collected for RNA extraction. Three independent biological replicates were used for each analysis. All samples were stored at −80 °C after being flash-frozen in liquid nitrogen for further experiments.

### 4.2. RNA Extraction and RT-qPCR Reactions

Total RNA of tea plant leaves and other tissues was isolated using the TIANGEN RNAprep Pure Plant Kit (Beijing, China), according to the manufacturer’s instructions. Complementary DNA (cDNA) templates for quantitative RT-PCR (RT-qPCR) were prepared using a TaKaRa PrimeScript™ RT reagent kit (Kusatsu, Japan) with 1 μg of total RNA according to the manufacturer’s protocol. cDNA was amplified by PCR using a Roche Light Cycler 480 (Basel, Switzerland) with SYBR Green I Master Mix of Roche (Basel, Switzerland). Two technical replicates and three biological replicates were performed. *CsPTB* was used as a reference gene in the expression level analysis using the formula 2^−ΔCt^ or 2^−ΔΔCt^ [42]. All RT-qPCR primer sequences are listed in Table S1. 

### 4.3. Sequence Alignment and Phylogenetic Tree Analysis

RT-PCR was used to amplify the CDS of *CsFtsH5* from the cDNA of ‘Longjing 43’ leaves. Primers are listed in Table S2. The molecular weight of the CsFtsH5 protein was computed by ProtParam (https://web.expasy.org/protparam/, accessed on 1 May 2022). AtFtsH and CsFtsH5 protein sequences were used to construct a phylogenetic tree. Alignment and phylogenetic tree assembly were performed using default settings and the neighbor-joining algorithm of MEGA version 7; 1000 bootstrap trials were used. The amino acid sequences of AtFtsH and CsFtsH5 are shown in Table S3. The AAA+ ATPase domain was identified using the NCBI Conserved Domain Database (https://www.ncbi.nlm.nih.gov/Structure/cdd/wrpsb.cgi, accessed on 15 August 2022). The homologous sequence of CsFtsH5 was analyzed using BLASTp against the NCBI database. The alignment of FtsH5 protein sequences was performed using DNAMAN. 

### 4.4. Subcellular Localization of CsFtsH5

To determine the subcellular localization of CsFtsH5, its open reading frame was cloned into a 35S-GFP vector which contained a C-terminal eGFP fragment [43]. The 35S-GFP and 35S-CsFtsH5-GFP vector plasmids were transformed into Agrobacterium strain GV3101 and transiently expressed in *Nicotiana benthamiana* expressing an H2B-RFP protein [28]. After 48 h, confocal microscopy images of the leaf cells were taken using a Zeiss LSM710 confocal laser scanning microscope (Oberkochen, Germany).

### 4.5. Down-Regulation of CsFtsH5 Gene in Tea Plant 

According to a previously characterized procedure for gene suppression in tea leaves [30], candidate AsODNs were obtained using Soligo software 2.2 with *CsFtsH5* as the input sequence. AsODNs and sODNs were synthesized by Youkang Biosystems Company (Zhejiang, China) (Table S4). To suppress the expression of *CsFtsH5* in ‘Longjing 43’ tea plant leaves, tea plants were grown under dark conditions for 20 min before injection, 1 mL of 25 μM *CsFtsH5*-AsODN solution was injected fully into the lower epidermis of the first mature leaf, and leaves injected with the equivalent sODN were used as controls.

### 4.6. Low-Temperature Treatment and Measurement of Fv/Fm and REL

*CsFtsH5*-AsODN plants and *CsFtsH5*-sODN plants were treated with cold stress (4 °C for 5 d, followed by 22 °C recovery for 2 d) and freezing stress (22 °C for 4.5 d, followed by −16 °C for 12 h, and then 22 °C recovery for 2 d) immediately after injection with oligonucleotides. *CsFtsH5*-sODN plants were used as controls. The phenotypes of the *CsFtsH5*-AsODN and *CsFtsH5*-sODN plants were recorded after recovery. Injected leaves were immediately collected after low-temperature treatment to measure the maximum photochemical efficiency of PSII (*Fv/Fm*) and REL. For REL measurement, 10 leaf discs punched using a 1 cm diameter hole puncher were collected and placed in a 50 mL tube with 10 mL of distilled water. The tubes were shaken at 200 rpm at 25 °C for 2 h. The conductivity of the solutions was measured at 25 °C using an Orion 5 Star conductivity meter of Thermo Fisher Scientific (Waltham, Massachusetts, USA) at 25 °C, as previously described [44]. Three independent biological replicates were used for REL determination. To measure *Fv/Fm*, *CsFtsH5*-AsODN and *CsFtsH5*-sODN plants were placed in the dark for 20 min, and *Fv/Fm* values were measured using FluorCam 7 of Photon Systems Instruments (Drasov, Czech Republic). The fluorescence parameters were integrated over the entire leaf area. Eight independent biological replicates were used for *Fv/Fm* determination.

### 4.7. Y2H

The full-length CDS of *CsFtsH5* and *CsCIPK11* were amplified from the cDNA of ‘Longjing 43’ tea plant leaves and cloned into pGADT7 (AD) and pGBKT7 (BD), respectively. According to the manufacturer’s protocol, the recombinant AD and BD vectors were co-transformed into yeast AH109 chemically competent cells of Weidi (Shanghai, China) in specified combinations. The AH109 cells co-expressing pGADT7-T with pGBKT7-p53 were used as the positive control (+). The AH109 cells co-expressing pGADT7-T with pGBKT7-lam and CsCIPK11-BD with the AD empty vector were used as the negative control. Positive transformants grown on DDO:SD/−Leu/−Trp medium (without tryptophan and leucine) were plated on the screening medium QDO: SD/−Ade/−His/−Leu/−Trp (without tryptophan, leucine, adenine, and histidine) to investigate the interactions. Serial dilutions (OD_600_ = 0.2, 0.02, and 0.002) were plated on DDO and QDO medium. Then, the plates were incubated at 30 °C for 7 days and recorded.

### 4.8. LCI Analysis

To perform the LCI assays, *CsFtsH5* and *CsCIPK11* CDSs were cloned into the C-terminal half of luciferase (C-Luc) and into the N-terminal half of luciferase (N-Luc). The plasmids were transformed into Agrobacterium strain GV3101-P19 of Weidi (Shanghai, China) and transiently expressed in *N. benthamiana*. The leaves co-expressing CsCIPK11-nLuc with cLuc, nLuc with CsFtsH-cLuc, as well as nLuc with cLuc, were used as the negative control. After 48 h, the LCI assay was performed using a chemiluminescence image analysis system of Tanon (Shanghai, China).

### 4.9. Dual-Luc Assays

The Dual-Luc assay was performed to analyze the stability of CsFtsH5, as previously described [45]. The coding regions of *CsFtsH5* were placed under the control of the 35S promoter, and the cassettes were inserted into pGreenII 0800-Luc, which contains the 35S-REN cassette, to generate pGreenII 0800-35S-CsFtsH5-Luc constructs. Subsequently, 35S-CsCIPK11 and 35S-empty vectors were co-expressed with 35S-CsFtsH5-Luc constructs in *N. benthamiana*. After 48 h, the LCI assay was performed using a chemiluminescence image analysis system of Tanon (Shanghai, China). These *N. benthamiana* specimens were then collected for the Dual-Luc assay using the Dual-Luciferase Reporter System of Promega (Madison, WI, USA) according to the manufacturer’s instructions, as previously described [46]. Six independent biological replicates were taken. The REN activity for each reaction was used as an internal control. 

### 4.10. Statistical Analysis

All experiments were performed with at least three independent biological replicates. Data represent the mean ± SEM of biological replicates. Data were statistically analyzed using *t*-tests and LSD tests performed using SPSS. Images were handled using GraphPad Prism 9, Adobe Photoshop, and Adobe Illustrator.

## Data Availability

Not applicable.

## Supplementary Materials

The following supporting information can be downloaded at: https://www.mdpi.com/article/10.3390/ijms24076288/s1.
