# Supplementary material for: CsCIPK11-Regulated Metalloprotease CsFtsH5 Mediates the Cold Response of Tea Plants
Source: Int J Mol Sci. 2023 Mar 27;24(7):6288. doi: 10.3390/ijms24076288 (PMC10094637; doi:10.3390/ijms24076288)
Supplement: Supplementary file 1 [file ijms-24-06288-s001.zip › Supplementary Figure S1.pdf]

Supplementary Figures

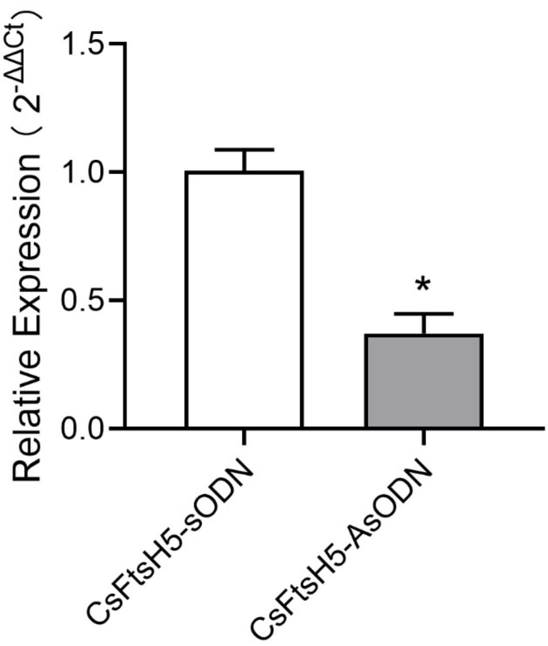

Figure S1. The expression of *CsFtsH5* in AsODN-CsFtsH5 and sODN-CsFtsH5 tea plant. Values was expressed relative to the expression levels of sODN-CsFtsH5 group using formula $2^{-\Delta\Delta Ct}$. *CsPTB* was used as a reference gene in expression analysis. Data are shown as the mean ± SEM (n=3). Asterisks indicate significant differences according to *t*-test (*$P < 0.05$).
