# Supplementary material for: CsCIPK11-Regulated Metalloprotease CsFtsH5 Mediates the Cold Response of Tea Plants
Source: Int J Mol Sci. 2023 Mar 27;24(7):6288. doi: 10.3390/ijms24076288 (PMC10094637; doi:10.3390/ijms24076288)
Supplement: Supplementary file 1 [file ijms-24-06288-s001.zip › Table S1 All RT-qPCR Primers used in this study.pdf]

Table S1 All RT-qPCR Primers used in this study

Primer name	Sequences
CsFtsH5-F	TTCTTAGGTCAACAGGTGTCT
CsFtsH5-R	TCTGCTTGGCCCTTTCGTATG
CsCIPK11-F	CATCGAGAAAGTTGAGGAGG
CsCIPK11-R	ACCATTCAGCCCTTCCAATTCC
CsCBF1-F	CGACTGAGACGACAATGCCGGA
CsCBF1-R	GTCACCAAGTTCCGCGTCATCA
CsCOR414-F	TTGGGTCCTGTTTGTTACTCTG
CsCOR414-R	AGCCCAAATACCATATTCACCC
CsCOR47 (TEA010666)-F	GAAATACGAGGCAGTGGTGA
CsCOR47 (TEA010666)-R	CTGGCCTGGTAGTTTCTCCT
CsCOR47 (TEA010673)-F	GAGAAATACGAGGCAGTGGTG
CsCOR47 (TEA010673)-R	TGGCCTGGTAGTTTCCCCT
CsMPK3-F	GGGTTCATTCGGAGCAAAGATG
CsMPK3-R	CAACAAGATCAACAGCTAATGG
CsPTB-F	GACAGCTGGGTTCCAGGCTT
CsPTB-R	AGCGACAGGGAGGAGAGGAT
