# Supplementary material for: CsCIPK11-Regulated Metalloprotease CsFtsH5 Mediates the Cold Response of Tea Plants
Source: Int J Mol Sci. 2023 Mar 27;24(7):6288. doi: 10.3390/ijms24076288 (PMC10094637; doi:10.3390/ijms24076288)
Supplement: Supplementary file 1 [file ijms-24-06288-s001.zip › Table S2 All RT-PCR Primers used in this study.pdf]

Table S2 All RT-PCR Primers used in this study

Primer name	Sequences
CsFtsH5-F	ATGGCTTCCACTAAACCCTT
CsFtsH5-R	TTAGGCAACAAATAGTTCAGC
CsCIPK11-F	ATGCCAGAGATCGAGCATGTCC
CsCIPK11-R	TTAGTTACCGGCGACCTGCTGC
35s-CsFtsH5-GFP-F	ggtacccggggatcctctagaATGGCTTCCACTAAACCCTT
35s-CsFtsH5-GFP-R	gcccttgctcaccatgtcgacGGCAACAAATAGTTCAGC
CsFtsH5-AD-F	gccatggaggccagtgaattcATGGCTTCCACTAAACCCTT
CsFtsH5-AD-R	cagctcgagctcgatggatccTTAGGCAACAAATAGTTCAGC
CsCIPK11-BD-F	atggccatggaggccgaattcACCTCGTTTGGGAACAGG
CsCIPK11-BD-R	ccgctgcaggtcgacggatccTTAGTTACCGGCGACCTGCTGC
CsFtsH5-cLuc-F	tacgcgtcccggggcggtaccATGGCTTCCACTAAACCCTT
CsFtsH5-cLuc-R	cagtcgacgcgttgtggatccTTAGGCAACAAATAGTTCAGC
CsCIPK11-nLuc-F	acgggggacgagctcggtaccATGCCAGAGATCGAGCATGTCC
CsCIPK11-nLuc-R	cagtcgacgcgttgtggatccGTTACCGGCGACCTGCTGC
35s-CsFtsH5-Luc-F	cgaattcctgcagcccggggATGGCTTCCACTAAACCC
35s-CsFtsH5-Luc-R	gctctagaactagtggatcGGCAACAAATAGTTCAGCTT
