# Supplementary material for: CsCIPK11-Regulated Metalloprotease CsFtsH5 Mediates the Cold Response of Tea Plants
Source: Int J Mol Sci. 2023 Mar 27;24(7):6288. doi: 10.3390/ijms24076288 (PMC10094637; doi:10.3390/ijms24076288)
Supplement: Supplementary file 1 [file ijms-24-06288-s001.zip › Table S3 The protein sequences used in this study.pdf]

Table S3 The protein sequences used in this study

CsFtsH5	MASTKPLLSSSFHGNQIFLSPPTPKTTKCSKLLIPQSILNRRPSNTTLKNLPSQATLAALLFSS<br>ITPQQTLALDTINPPNAPPPAIQAEAPKPIPSPFSQNLLMTAPKPQTQSDIPDGTQWRYSEFL<br>SAVKKGKVERVRFSKDGTALQLTAVDGRRATVTVPSDPDLIDILASNAVDISVAEGDSGN<br>GLFSFIGNLIFPFLAFAGLFLLFRRSQGGPGGPGGFGGPMDFGRSKSKFQEVPETGVSFSDV<br>AGADQAKLELQEVVDFLKNPDKYTALGAKIPKGCLLVGPPGTGKTLLARAVAGEAGVPF<br>FSCAASEFVELFVGVGASRVRDLFEKAKSKAPCIVFIDEIDAVGRQRGAGLGGGNDEREQ<br>TINQLLTEMDGFSGNSGVIVLAATNRPDVLDSALLRPGRFDRQVTVDRPDVAGRVRILQV<br>HSRGKALAKDVDFEKISRRTPGFTGADLQNLMNEAAILAARRDLKEISKDEISDALERIIAG<br>PEKKNAVVSEAKKKLVAYHEAGHALVGALMPEYDPVAKISIIPRGQAGGLTFFAPSEERL<br>ESGLYSRSYLENQMAVALGGRVAEEVIFGDDNVTTGASNDFMQVSRVARQMVERFGFSK<br>KIGQVAIGGSGGNPFLGQQVSSQKDYSMATADVVDTEVRELVEKAYERAKQIITTHIDILH<br>KLALLLVEKETVDGEEFMSLFIDGKAELFVA
AtFtsH1	MASNSLLRSSSNFFLGSHIIISSPTPKTTRKPSFPFSFVSRAKYQITRSSQDENSPNGKPNSPFS<br>SQVALAAILLSSISSSPLALAVVDEPASPSVVIESQAVKPSTPSPLFIQNEILKAPSPKSSDLPE<br>GSQWRYSEFLNAVKKGKVERVRFSKDGSVVQLTAVDNRRASVIVPNDPDLIDILAMNGV<br>DISVSEGESSGNDLFTVIGNLIFPLLAFGGLFLLFRRAQGGPGGGPGGLGGPMDFGRSKSKF<br>QEVPETGVSFADVAGADQAKLELQEVVDFLKNPDKYTALGAKIPKGCLLVGPPGTGKTL<br>LARAVAGEAGVPFFSCAASEFVELFVGVGASRVRDLFEKAKSKAPCIVFIDEIDAVGRQRG<br>AGMGGGNDEREQTINQLLTEMDGFSGNSGVIVLAATNRPDVLDSALLRPGRFDRQVTVD<br>RPDVAGRVKILQVHSRGKALGKDVDFDKVARRTPGFTGADLQNLMNEAAILAARRELKE<br>ISKDEISDALERIIAGPEKKNAVVSEEKKRLVAYHEAGHALVGALMPEYDPVAKISIIPRGQ<br>AGGLTFFAPSEERLESGLYSRSYLENQMAVALGGRVAEEVIFGDENVTTGASNDFMQVSR<br>VARQMIERFGFSKKIGQVAVGGPGGNPFMGQQMSSQKDYSMATADIVDAEVRELVEKA<br>YKRATEIITTHIDILHKLAQLLIEKETVDGEEFMSLFIDGQAELYIS
AtFtsH2	MNFQMAASSACLVGNGLSVNTTTKQRLSKHFSGRQTSFSSVIRTSKVNVVKASLDGKKK<br>QEGRRDFLKILLGNAGVGLVASGKANADEQGVSSSRMSYSRFLEYLDKDRVNKVDLYEN<br>GTIAIVEAVSPELGNRVERVRVQLPGLSQELLQKLRAKNIDFAAHNAQEDQGSVLFNLIGN<br>LAFPALLIGGLFLLSRRSGGGMGGPGGPGNPLQFGQSKAKFQMEPNTGVTFDDVAGVDE<br>AKQDFMEVVEFLKKPERFTAVGAKIPKGVLLIGPPGTGKTLLAKAIAGEAGVPFFSISGSEF<br>VEMFVGVGASRVRDLFKKAKENAPCIVFVDEIDAVGRQRGTGIGGGNDEREQTLNQLLT<br>EMDGFEGNTGVIVVAATNRADILDSALLRPGRFDRQVSVDVPDVKGRTDILKVHAGNKK<br>FDNDVSLEIIAMRTPGFSGADLANLLNEAAILAGRRARTSISSKEIDDSIDRIVAGMEGTVM<br>TDGKSKSLVAYHEVGHAVCGTLTPGHDAVQKVTLIPRGQARGLTWFIPSDDPTLISKQQL<br>FARIVGGLGGRAAEEIIFGDSEVTTGAVGDLQQITGLARQMVTTFGMSDIGPWSLMDSSA<br>QSDVIMRMMARNSMSEKLAEDIDSAVKKLSDSAYEIALSHIKNNREAMDKLVEVLLEKE<br>TIGGDEFRAILSEFTEIPPENRVPSSTTTTPASAPTPAAV
AtFtsH3	MTMIFFSKLNRSISRSKGFLYGGGVRSAARLLTSPGLEAASVNEVEGGLGFIRRHFASL<br>ASRKGLVNNDLIGVFANPRLRRFFSDEAPKKKNYENYFPKDKQEPKSDQKSEHKEGS<br>EKNENENVGDMFMNRFQNLLIPLLALAVFFSTFSFGSGEQQQISFQEFKNKLLEPGLV<br>DHIDVSNKSVAKVYVRSTPKDQQTTDVVHGNGNGIPAKRTGGQYKYYFNIGSVDSFE<br>EKLEEAQEALGVDRHEYVPVTYVSEMVWYQEFMRFAPTLLLLGTLIYGARRMQGGL<br>GVGGTGGKNGRGIFNIGKATITRADKHSKNKIYFKDVAGCDEAKQEIMEFVHFLKNP

	KKYEDLGAKIPKGALLVGPPGTGKTLLAKATAGESGVPFLSISGSDFMEMFVGVGPSR<br>VRHLFQEARQAAPSIIFIDEIDAIGRARGRGGLGGNDERESTLNQLLVEMDGFGTTAG<br>VVVLAGTNRPDILDKALLRPGRFDRQITIDKPDIKGRDQIFKIYLKKIKLDHEPSYYSQ<br>RLAALTPGFAGADIANVCNEAALIAARHEGATVTMAHFESAIDRVIGGLEKKNRVISK<br>LERRTVAYHESGHAVVGWFLEHAEPLLKVTIVPRGTAALGFAQYVPNENLLMTKEQL<br>FDMTCMTLGGRAAEQVLIGKISTGAQNDLEKVTKMTYAQVAVYGFSDKVGLLSFPPR<br>DDGYDFSKPYSNKTGAIIDEEVRDWVAKAYERTVELVEEHKVKVAEIAELLLEKEVLH<br>QDDLLKILGERPFKSAEVTNYDRFKSGFEETEKDSAATPTVEPVVDDGAPPPFEPQVV<br>PT
AtFtsH4	MAWRRIITKVSSHERELSSLRSLLVRAYSSFPRVGVTGAVGGGGASLPRTRFQSSYVGS<br>FARRVRDREEVNEVAHLRELIRRNDPEAVIRMFESQPSLHANASALSEYIKALVKVDR<br>LDQSELVRTLQRGIAGVAREEETFGGLGAFRNVGKPTKDGVLGTASAPIHTISTERTHF<br>KEQLWSTIRTIGVGFLLISGIGALIEDRGIGKGLGLHEEVQPSMDSSTKFSDVKGVDEA<br>KAELEEIVHYLRDPKRFTRLGGKLPKGVLLVGPPGTGKTMLARAIAGEAGVPFFSCSG<br>SEFEEMFVGVGARRVRDLFSAAKKCSPCIIFIDEIDAIGGSRNPKDQQYMKMTLNQML<br>VELDGFKQNEGIIVVAATNFPESLDKALVRPGRFDRHIVVPNPDVEGRRQILESHMSK<br>VLKAEDVDLMIIARGTPGFSGADLANLVNVAALKAAMDGSKDVTMSDLEFAKDRIM<br>MGSERKSAVISDESRKLTAFHEGGHALVAIHTEGALPVHKATIVPRGMALGMVSQLPD<br>KDETSISRKQMLARLDVCMGGRVAEELIFGESEVTSGASSDLEQATKLARAMVTKFG<br>MSKEVGLVAHNYDDNGKSMSTETRLLIESEVKQLLEKAYNNAKTILTVYNKELHALA<br>NALLQHETLSGKQIKELLTDLNSPLLQKRQEVVTKQSNPVPPSTPSSASSAAAAAAAA<br>AAAAAAAAATAATKGKDMAPVS
AtFtsH5	MATTSSNPLLLSSNFLGSQIIISAPTPKTTTKSLPFSVISRKRYQISQSEKLMKSLPSQAA<br>LAALLFSSSSPQALAVNEPVQPPAPTITAEAQSPNLSTFGQNVLMTAPNPQAQSSDLPD<br>GTQWRYSEFLNAVKKGKVERVKFSKDGSVLQLTAVDNRRATVIVPNDPDLIDILAMN<br>GVDISVSEGEGGNGLFDFIGNLLFPLLAFGGLFYLFRGGQGGAGGPGGLGGPMDFGR<br>SKSKFQEVPETGVTFGDVAGADQAKLELQEVVDFLKNPDKYTALGAKIPKGCLLVGP<br>PGTGKTLLARAVAGEAGVPFFSCAASEFVELFVGVGASRVRDLFEKAKSKAPCIVFID<br>EIDAVGRQRGAGMGGGNDEREQTINQLLTEMDGFSGNSGVIVLAATNRPDVLDSALL<br>RPGRFDRQVTVDRPDVAGRVQILKVHSRGKAIGKDVDYEKVARRTPGFTGADLQNL<br>MNEAAILAARRELKEISKDEISDALERIIAGPEKKNAVVSEEKKRLVAYHEAGHALVG<br>ALMPEYDPVAKISIIPRGQAGGLTFFAPSEERLESGLYSRSYLENQMAVALGGRVAEEVI<br>FGDENVTTGASNDFMQVSRVARQMVERFGFSKKIGQVAVGGAGGNPFLGQSMSSQK<br>DYSMATADVVDAEVRELVEKAYVRAKEIITTQIDILHKLAQLLIEKETVDGEEFMSLFI<br>DGQAELYVS
AtFtsH6	MKMASSSSALSFPLSNIPTCSKKSQQFQKPASLSKSSHTHKPSLKTQILHHKFTKRNLL<br>SLTTALGFTSALGTVLAHPAKAEPEAPIEATSNRMSYSRFLQHLKENEVKKVDLIENGT<br>VAIVEISNPVVGKIQRVRVNLPGLPVDLVREMKEKNVDFAAHPMNVNWGAFLLNFLG<br>NLGFPLILLVSLLLTSSSRRNPAGPNLPFGLGRSKAKFQMEPNTGITFEDVAGVDEAKQ<br>DFEEIVEFLKTPEKFSALGAKIPKGVLLTGPPGTGKTLLAKAIAGEAGVPFFSLSGSEFI<br>EMFVGVGASRARDLFNKAKANSPCIVFIDEIDAVGRMRGTGIGGGNDEREQTLNQILT<br>EMDGFAGNTGVIVIAATNRPEILDSALLRPGRFDRQVSVGLPDIRGREEILKVHSRSKK<br>LDKDVSLSVIAMRTPGFSGADLANLMNEAAILAGRRGKDKITLTEIDDSIDRIVAGME<br>GTKMIDGKSKAIVAYHEVGHAICATLTEGHDPVQKVTLVPRGQARGLTWFLPGEDPTL

	VSKQQLFARIVGGLGGRAAEDVIFGEPEITTGAAGDLQQVTEIARQMVTMFGMSEIGP WALTDPAVKQNDVVLRMLARNSMSEKLAEDIDSCVKKIIGDAYEVAKKHVRNNREAI DKLVDVLLEKETLTGDEFRAILSEYTDQPLNTDGDVRIRINDLISV
AtFtsH7	MTTTFEFLQPRIHGFATCCSSNSLLYSKASRFFNDRCRVYRQNPNRFVSNSITLPLQKK QVTVLRNHERFNLWDGFSRKKSRLVVNCQEDDQNESSSEEEESSQSTPAKSERKREK KEDKVWWSKGKKWQWQPIIQAQGIGVLLLQLSVVMFVMRLLRPGIPLPGSEPRIQTT FVSVPYSEFLSKVNSNQVQKVEVDGVQVLFKLRDDGKWQESETSRLSQSSESLLRTV APTKRVVYSTTRPGDIKTPYEKMLGNNVEFGSPEKRSGGFFNSALIALFYIAVLAGLIR FPVSFSTSSTGQLRTRKAGGPDGGKVSGGGETITFADVAGVDEAKEELEEIVEFLRNPE KYVRLGARPPRGVLLVGLPGTGKTLLAKAVAGEAEVPFISCSASEFVELYVGMGASRV RDLFARAKKEAPSIIFIDEIDAVAKSRDGKFRMGSNDEREQTLNQLLTEMDGFDSNSAV IVLGATNRADVLDPALRRPGRFDRVVTVETPDKIGRESILRVHVSKKELPLGDDVNLG SIASMTTGFTGADLANLVNEAALLAGRKNKTNVEKIDFIQAVERSIAGIEKKSARLKG NEKAVVARHEAGHAVVGTAVANLLTGQPRVEKLSILPRTGGALGFTYIPPTSEDRYLLFI DELLGRLVTLLGGRAAEEVVYSGRISTGAFDDIRRATDMAYKAVAEYGLNQKIGPVSV ATLSGGGIDDSGGSPWGRDQGKLVDLVQKEVTILLQSALDVALSVVRANPDVLEGLG AQLEEKEKVEGEELQKWLSMVVAPEELAVFVEGKQELLLPAQASSS
AtFtsH8	MAASSACLLGNGLSVYTTKQRFQKLGLDRTSKVTVVKASLDEKKHEGRRGFFKLLL GNAAAGVGLLASGNANADEQGQGVSSSRMSYSRFLEYLDKGRVEKVDLYENGTIAI VEAVSPELGNRIQRVRVQLPGLSQELLQKLRAKNIDFAAHNAQEDQGSPILNLIGNLAF PVILIGGLFLLSRRSSGGMGGPGGPGFPLQIGQSKAKFQMEPNTGVTFDDVAGVDEAK QDFMEVVEFLKKPERFTAVGARIPKGVLLVGPPGTGKTLLAKAIAGEAGVPFFSISGSE FVEMFVGVGASRVRDLFKKAKENAPCIVFVDEIDAVGRQRGTGIGGGNDEREQTLNQ LLTEMDGFEGNTGVIVVAATNRADILDSALLRPGRFDRQVSVDVPDVKGRTDILKVHS GNKKFESGVSLEVIAMRTPGFSGADLANLLNEAAILAGRRGKTAISSKEIDDSIDRIVA GMEGTVMTDGKSKSLVAYHEVGHAICGTLTPGHDAVQKVTLIPRGQARGLTWFIPSD DPTLISKQQLFARIVGGLGGRAAEEVIFGESEVTTGAVSDLQQITGLAKQMVTTFGMS EIGPWSLMDSSEQSDVIMRMMARNSMSEKLANDIDTAVKTLSDKAYEIALSQIRNNRE AMDKIVEILLEKETMSGDEFRAILSEFTEIPPENRVASSTSTSTPTPASV
AtFtsH9	MTSIELLSPLIHDKFRFSTCCSTSSLLYLHASSFFRDRSFGFRQNPNRFVSNSSIQLPQSV PGSINQERFNLWQGFSRKKSTSSSRTIVNCQEGDQKASSSEGEGKTNKDKGRKQGKN ELWWSKGKKWQWKPIIQAQEIGVMLLQLGIVMFVVRLLRPGIPLPGSEPRTQTTFMS VPYSDFLSKVNNDEVQKVEVDGFHVLFKLKDDGNLQESETSSSSIKLSESSETMLRSV APTKRVVYSTTRPRDIKTPYEKMLENNVEFGSPDKRSGGFFNSGLIVLFYIAVLAGLLH RFPVNFSQSTTGQLRTRKSGGPGGGKVSGDGETITFADVAGVDEAKEELEEIVEFLKN PDRYVRLGARPPRGVLLVGLPGTGKTLLAKAVAGESDVPFISCSASEFVELYVGMGAS RVRDLFARAKKEAPSIIFIDEIDAVAKSRDGKFRMVSNDEREQTLNQLLTEMDGFDSSS AVIVLGATNRADVLDPALRRPGRFDRVVTVESPDKVGRESILKVHVSKKELPLGDDVN LASIASMTTGFTGADLANLVNEAALLAGRKSKMTVDKIDFIHAVERSIAGIEKKTARL KGSEKAVVARHEAGHAVVGTAVASLLSGQSRVEKLSILPRSGGALGFTYIPPTHEDRYL LFIDELHGRLVTLLGGRAAEEVVYSGRISTGALDDIRRATDMAYKAVAEYGLNEKIGP VSVATLSAGGIDDSGGSPWGRDQGHLVDLVQREVTNLLQSALDVALTVVRANPDVLE GLGAQLEDEEKVEGEELQKWLNRVVPSEELAVFIKGKQTALLPAQASSS
AtFtsH10	MIFSKLGSSLARSSRSKGFVYGGGVRSAVFNQGRLRAPQNLEAAVNQVDGGLGFLRR

	HFASFAARKGLEAGDLSRAFANPRLRRFFSSQTPKKKNYENYYPKDSKKAPKNEQKS ESRDGSKKNENENAGDAFSNEYQNMLIPLMAIALILSTFSLGSREQQQISFQEFKNKLL EAGLVDHIDVSNKEVAKVYVRSSPKSQTTEEVVQGPGNGVPAKGRGGQYKYYFNIGS VESFEEKLEEAQEAIGVNSHDFVPVTYVSETIWYQELLRFAPTLLLVATLIFGARRMQG GLGGLGGPGGKAGRGIFNIGKAQITRADKNSKNKIYFKDVAGCEEAKQEIMEFVHFL QNPKKYEDLGAKIPKGALLVGPPGTGKTLLAKATAGESAVPFLSISGSDFMEMFVGVG PSRVRNLFQEARQCAPSIIFIDEIDAIGRARGRGGFSGGNDERESTLNQLLVEMDGFGT TAGVVVLAGTNRPDILDKALLRPGRFDRQITIDKPDIKGRDQIFQIYLKKIKLDHEPSY YSQRLAALTPGFAGADIANVCNEAALIAARHEGATVTMAHFDSAIDRVIGGLEKKNR VISKLERRTVAYHESGHAVAGWFLEHAEPLLKVTIVPRGTAALGFAQYVPNENLLMTK EQLFDMTCMTLGGRAAEQVLIGRISTGAQNDLEKVTKMTYAQVAVYGFSDKIGLLSF PQREDEFSKPYSNRTGAMIDEEVREWVGKAYKRTVELIEEHKEQVAQIAELLLEKEVL HQDDLTKVLGERPFKSGETTNYDRFKSGFEESEKESQKESVPVKPVEDDGIPPLEPQV VPT
AtFtsH11	MSSSTLQASLFLRPPLHTSSFKLYPCLFSSSSLSFCPQSLSSFYRLSSVLHNSRFRPLPCS LRQDNVASDSDFIPKDSAFEVTDSAESNRLVSDTEVSELETNDRFVGGEETKSGGEEA EVSNGVTEGKEEDQKKSKFRIVVLMMALWAAIKRAIEKVMEWEWLSWWPFSRQEK RLEKLIAEADANPKDAALQGALLAELNKHIPEAVVQRFEQREHTVDSRGVAEYIRALV ITNAISEYLPDEQTGKPSSLPALLQELKHRASGNMDESFVNPGISEKQPLHVTMVNPK VSNKSRFAQELVSTILFTVAVGLVWIMGAAALQKYIGSLGGIGTSGVGSSSSYSPKELN KEITPEKNVKTFKDVKGCDDAKQELEEVVEYLKNPSKFTRLGGKLPKGILLTGAPGT GKTLLAKAIAGEAGVPFFYRAGSEFEEMFVGVGARRVRSLFQAAKKKAPCIIFIDEIDA VGSTRKQWEGHTKKTLHQLLVEMDGFEQNEGIIVMAATNLPDILDPALTRPGRFDRHI VVPSPDVRGREEILELYLQGKPMSEDVDVKAIARGTPGFNGADLANLVNIAAIKAAVE GAEKLSSEQLEFAKDRIVMGTERKTMFVSEDSKKLTAYHESGHAIVALNTKGAHPIHK ATIMPRGSALGMVTQLPSNDETSVSKRQLLARLDVCMGGRVAEELIFGLDHITTGASS DLSQATELAQYMVSSCGMSEAIGPVHIKERPSSDMQSRIDAEVVKLLREAYERVKSLL KRHEKQLHTLANALLEYETLTAEDIKRILLPKQEGEKFEEQQQEEGDLVLA
AtFtsH12	MEIAISYKPNPLISSSTQLLKRSKSFGLVRFPAKYGLGATRKKQLFRVYASESSSGSSSN SDGGFSWVRLAQSIRLGAERIGEKIGESVKTEIGFDSEEASGRVNEYVARVKDSVHKG HHELTRFKNETVPSFIDWNKWEHWKDIRNWDGKRVAALFIYAFALLLSCQRVYVAIQ APRVERERRELTESFMEALIPEPSPGNIEKFKRNMWRKATPKGLKLKRFIEAPDGTLVH DSSYVGENAWDDDLETTEGSLKKIIGRNARIQTEAKKKLSQDLGVSGEIGDSVGNWR ERLATWKEMLEREKLSEQLNSSAAKYVVEFDMKEVEKSLREDVIGRTSETEGTRALW ISKRWWRYRPKLPYTYFLQKLDSSEVAAVVFTEDLKRLYVTMKEGFPLEYIVDIPLDP YLFETICNAGVEVDLLQKRQIHYFMKVFIALLPGILILWFIRESAMLLLITSKRFLYKKY NQLFDMAYAENFILPVGDVSETKSMYKEVVLGGDVWDLLDELMIYMGNPMQYYEK DVAFVRGVLLSGPPGTGKTLFARTLAKESGLPFVFASGAEFTDSEKSGAAKINEMFSIA RRNAPAFVFVDEIDAIAGRHARKDPRRRATFEALIAQLDGEKEKTGIDRFSLRQAVIFIC ATNRPDELDLEFVRSGRIDRRLYIGLPDAKQRVQIFGVHSAGKNLAEDIDFGKLVFRTV GFSGADIRNLVNEAAIMSVRKGRSYIYQQDIVDVLDKQLLEGMGVLLTEEEQQKCEQ SVSYEKKRLLAVHEAGHIVLAHLFPRFDWHAFSQLLPGGKETAVSVFYPREDMVDQG YTTFGYMKMQMVVAHGGRCAERVVFGDNVTDGGKDDLEKITKIAREMVISPQSARL GLTQLVKKIGMVDLPDNPDGELIKYRWDHPHVMPAEMSVEVSELFTRELTRYIEETEE

	LAMNALRANRHILDLITRELLEKSRITGLEVEEKMKDLSPLMFEDFVKPFQINPDDEEL LPHKDRVSYQPVDLRAAPLHRS
AcFtsH5	MASTTTNPLFSSRFLGNQILLSPPTPNTTKFPSLSRRKFLVPQSILNRKPNHLNSIPSQAT LAALIFSSITPQALAVDTANPPNPPPVISAEAPKPIPSNSSSPFSQNLLLTAPKPQTQTTSD LPEGSQWRYSEFLNAVKKGKVERVRFSKDGSALQLTAVDGRRASVIVPNDPDLIDILA MNGVDISVSEGDSGNGLFSFIGNLLFPFLAFAGLFFLFRRSQGGPGGPGGLGGPMDFG RSKSKFQEVPETGVTFADVAGADQAKLELQEVVDFLKNPDKYTALGAKIPKGCLLVG PPGTGKTLLARAVAGEAGVPFFSCAASEFVELFVGVGASRVRDLFEKAKSKAPCIVFID EIDAVGRQRGAGLGGGNDEREQTINQLLTEMDGFSGNSGVIVLAATNRPDVLDSALL RPGRFDRQVTVDRPDVAGRVKILQVHSRGKALAKDVDFEKISRRTPGFTGADLQNLM NEAAILAARRDLKEISKDEISDALERIIAGPEKKNAVVSEEKKKLVAYHEAGHALVGAL MPEYDPVAKISIIPRGQAGGLTFFAPSEERLESGLYSRSYLENQMAVALGGRVAEEVIFG EDNVTTGASNDFMQVSRVARQMVERFGFSKKIGQIAIGGPGGNPFLGQQMSSQKDYS MATADIVDAEVRELVEKAYSRSKQIITTHIDILHKLAQLLIEKETVDGEEFMSLFIDGKA ELFVA
NtFtsH5	MANSLLSSNFMGSQIFVSPPTPKTTKYFHFHSKRKSLIPQSILNKKPNSDNSKNIPSKAA LAALLFSSITPHAYALDNTTPTVPTPRVIQAEAANPTTSNPFSQNIILNAPKPQAQTNPE LPEVSQWRYSEFLNAVKKGKVERVRFSKDGSALQLTAVDGRRATVTVPNDPDLIDILA MNGVDISVSEGDSAGNGLFNLIGNLFPFIAFAGLFYLFQRSQGGPGGPGGLGGPMDFG RSKSKFQEVPETGVTFADVAGADQAKLELQEVVDFLKNPDKYTALGAKIPKGCLLVG PPGTGKTLLARAVAGEAGVPFFSCAASEFVELFVGVGASRVRDLFEKAKSKAPCIVFID EIDAVGRQRGAGMGGGNDEREQTINQLLTEMDGFSGNSGVIVLAATNRPDVLDSALL RPGRFDRQVTVDRPDVAGRIKILQVHSRGKALTKDVDFEKIARRTPGYTGADLQNLM NEAAILAARRELKEISKDEISDALERIIAGPEKKNAVVSDEKKKLVAYHEAGHALVGAL MPEYDPVAKISIIPRGQAGGLTFFAPSEERLESGLYSRSYLENQMAVALGERVAEEVIFG QDNVTTGASNDFMQVSRVARQMVERLGFSKKIGQVAIGGGGGNPFLGQQMSTQKDY SMATADVVDAEVRELVERAYERATEIITTHIDILHKLAQLLIEKETVDGEEFMSLFIDGK AELYISWVSKEED
OsFtsH1	MAPPCSISSASHLLITASLPKPSLRPPRLPHPKPLPAALLALAAAAPTLPALADVPAPPPS PTQDVQVLEAPSPAANPFSNALLTAPKPTSSAAADLPEGAQWRYSEFLSAVKKGKVER VRFSKDGGLLQLTAIDGRRATVVVPNDPDLIDILATNGVDISVAEGDAAGPGGFLAFV GNLLFPFLAFAGLFFLFRRAQGGPGAGPGGLGGPMDFGRSKSKFQEVPETGVTFVDV AGADQAKLELQEVVDFLKNPDKYTALGAKIPKGCLLVGPPGTGKTLLARAVAGEAG VPFFSCAASEFVELFVGVGASRVRDLFEKAKAKAPCIVFIDEIDAVGRQRGAGLGGGN DEREQTINQLLTEMDGFAGNSGVIVLAATNRPDVLDAALLRPGRFDRQVTVDRPDVA GRVKILEVHSRGKALAKDVDFEKIARRTPGFTGADLQNLMNEAAILAARRDLKEISK DEISDALERIIAGPEKKNAVVSEEKRRLVAYHEAGHALVGALMPEYDPVAKISIIPRGQ AGGLTFFAPSEERLESGLYSRSYLENQMAVALGGRVAEEVIFGQENVTTGASNDFMQV SRVARQMVERFGFSKKIGQVAIGGPGGNPFLGQQMSSQKDYSMATADVVDAEVRELV EKAYSRATQIITTHIDILHKLAQLLMEKETVDGEEFMSLFIDGQAELFVA
PtFtsH5	MASSTRNPLLSSDLFGSKVLLSPPTPKTTKSSFLLSKNLLKIVNKKKSGSFKSLQSQATI AAALLFSSLTPQALAIDNPAPPPTPPPVIEALPTKPSSPFAQNLLVTAPKPQSESTSDLPE GSQWRYSEFLNAVKKGKVERVRFSKDGSTLQLSAVDGRRASVVVLNDPDLIDILARS GVDISVSEGDSGNGFFNVIGSLFFPILAVAGLFLLFRRVQGGPGGPGGLGGPMDFGRSK

	SKFQEVPETGVSFADVAGADQAKLELQEVVDFLKNPDKYTALGAKIPKGCLLVGSPG TGKTLLARAVAGEAGVPFFSCAASEFVEMFVGVGASRVRDLFEKAKSKAPCIVFIDEI DAVGRQRGAGLGGGNDEREQTINQLLTEMDGFSGNSGVIVLAATNRPDVLDSALLRP GRFDRQVTVDRPDIAGRVKILQVHSRGKALAKDVDFEKIARRTPGFTGADLQNLMNE AAIVAARRDLKEISKDEISDALERIIAGPEKKNAVVSDEKKRLVAYHEAGHALVGALM PEYDPVAKISIIPRGQAGGLTFFAPSEERLESGLYSRSYLENQMAVALGGRVAEEVIFGQ DNVTTGASSDFMQVSRVARQMVERFGFSKKIGQVAIGGSGGNPFLGQQMSSQKDYS MATADVVDTEVRELVETAYSRAKQIMTTHIDILHKLAQLLIEKETVDGEEFMSLFIDGK AELYIA
VvFtsH5	MASSSTTNPLLSSTLFGNRISTPKTTKSSIPLQFFSRRRFEVTRSILNGKPRSELPSKAAL AAIIASSLAPQALAVDNATPPVPPPVIEAQPTKPSPSNSSPFAQNLLLTAPKPQTQSDLPE GSQWRYSEFLNAVKKGKVERVRFSKDGSALQLTAVDGRRATVIVPNDPDLIDILAMN GVDISVSEGDSGNGLFNFIGNLLFPFLAFAGLFFLFRRAQGGPGGPGGLGGPMDFGRS KSKFQEVPETGVTFADVAGADQAKLELQEVVDFLKNPDKYTALGAKIPKGCLLVGPP GTGKTLLARAVAGEAGVPFFSCAASEFVELFVGVGASRVRDLFEKAKSKAPCIVFIDEI DAVGRQRGAGLGGGNDEREQTINQLLTEMDGFSGNSGVIVLAATNRPDVLDSALLRP GRFDRQVTVDRPDVAGRVKILQVHSRGKALAKDVDFEKIARRTPGFTGADLQNLMN EAAILAARRDLKEISKDEISDALERIIAGPEKKNAVVSDEKKKLVAYHEAGHALVGAL MPEYDPVAKISIIPRGQAGGLTFFAPSEERLESGLYSRSYLENQMAVALGGRVAEEVIFG EDNVTTGASNDFMQVSRVARQMVERFGFSKKIGQVAIGGPGGNPFLGQQMSSQKDY SMATADIVDAEVRELVEKAYSRAKQIMTTHIDILHKLAQLLIEKETVDGEEFMSLFIDG KAELFVA
ZmFtsH5	MAPPSVSASHLLITASLPKPKPSSLRPPRLPLAKPLPASAALLALAASPALAADAPAPAP APTPAPAPELQAEAPTPTVNPFSSSLLTAPKPSAAASDLPEGAQWRYSEFLSAVKRGKV ERVRFSKDGGLLQLTAVDGRRATVVVPNDPDLIDILATNGVDISVSEGESAGPGGFVAF VGNLLFPFIAFAGLFFLFRRAQGGPGAGPGGLGGPMDFGRSKSKFQEVPETGVTFLDV AGADQAKLELQEVVDFLKNPDKYTALGAKIPKGCLLVGPPGTGKTLLARAVAGEAG VPFFSCAASEFVELFVGVGASRVRDLFEKAKAKAPCIVFIDEIDAVGRQRGAGLGGGN DEREQTINQLLTEMDGFAGNSGVIVLAATNRPDVLDSALLRPGRFDRQVTVDRPDVA GRVKILEVHSRGKALAKDVDFDKIARRTPGFTGADLQNLMNEAAILAARRDLKEISK DEISDALERIIAGPEKKNAVVSEEKKRLVAYHEAGHALVGALMPEYDPVAKISIIPRGQ AGGLTFFAPSEERLESGLYSRSYLENQMAVALGGRVAEEVIFGQDNVTTGASNDFMQV SRVARQMVERFGFSKKIGQVAIGGPGGNPFLGQQMSSQKDYSMATADVVDAEVRELV EKAYSRARQIITTHIDILHKLAQLLIEKETVDGEEFMSLFIDGQAELFVA
