# Supplementary material for: CsCIPK11-Regulated Metalloprotease CsFtsH5 Mediates the Cold Response of Tea Plants
Source: Int J Mol Sci. 2023 Mar 27;24(7):6288. doi: 10.3390/ijms24076288 (PMC10094637; doi:10.3390/ijms24076288)
Supplement: Supplementary file 1 [file ijms-24-06288-s001.zip › Table S4 AsODNs and sODNs primer sequences in this study.pdf]

Table S4 AsODNs and sODNs primer sequences in this study

Primer name	Sequences
sODN	GGCGGCTAACGCTTCGA
CsFtsH5-AsODN-1	GGGTTAATGGTGTCTAG
CsFtsH5-AsODN-2	AGGGCAGTTCCGTCTTT
CsFtsH5-AsODN-3	GTGTACTTATCTGGGTT
CsFtsH5-AsODN-4	CATCCGGTCTGTTCGTT
CsFtsH5-AsODN-5	ATGGGTTGTGATGATCT
